# Comparative Genotypic and Phenotypic Characterisation of Methicillin-Resistant *Staphylococcus aureus* ST398 Isolated from Animals and Humans

**DOI:** 10.1371/journal.pone.0040458

**Published:** 2012-07-11

**Authors:** Dorota M. Jamrozy, Mark D. Fielder, Patrick Butaye, Nick G. Coldham

**Affiliations:** 1 Department of Bacteriology, Animal Health and Veterinary Laboratories Agency, New Haw, United Kingdom; 2 School of Life Sciences, Faculty of Science, Engineering and Computing, Kingston University, London, United Kingdom; 3 Unit of General Bacteriology, Veterinary and Agrochemical Research Centre, Brussels, Belgium; 4 Department of Pathology, Bacteriology and Poultry Diseases, Ghent University, Ghent, Belgium; National Institutes of Health, United States of America

## Abstract

The high prevalence of methicillin-resistant *Staphylococcus aureus* (MRSA) ST398 among pigs in certain European countries and North America and its occurrence in other animal species raises a question concerning the molecular mechanisms mediating the success of this lineage. In this study a panel of *S. aureus* strains belonging to sequence type (ST) 5 (n = 4), ST8 (n = 5), ST15 (n = 5), ST22 (n = 8), clonal complex (CC) 30 (n = 8), CC97 (n = 8), CC130 (n = 4), CC151 (n = 4) and ST398 (n = 18) were screened by DNA microarray and PCR for the carriage of virulence and antimicrobial resistance genes. Isolates belonging to the same sequence type/clonal complex (ST/CC) were found to share similar virulence gene profiles. The ST398 lineage displayed the lowest content of virulence genes, which consisted mainly of genes detected among the majority or all of the analysed lineages. All MRSA ST398 isolates lacked accessory virulence genes that were detected in other ST/CC. In contrast to virulence genotype, the antimicrobial resistance genes profiles varied between isolates belonging to the same ST/CC and profile similarities could be observed for isolates from different lineages. MRSA ST398 isolates in particular displayed significant diversity and high content of antimicrobial resistance genes. This was comparable with certain MRSA belonging to other sequence types particularly the equine MRSA ST8. The apparent lack of significant virulence genes among MRSA ST398 strains, demonstrates that the lineage features a unique genetic background but no ST398-specific virulence markers could be identified.

## Introduction

Molecular epidemiological studies on *S. aureus* in humans has shown that the population consists of several prevalent lineages [1], which are best illustrated by the hospital- and community-acquired methicillin-resistant *S. aureus* strains where specific clonal complexes are dominant. The existence of such lineages among animal-associated isolates has also been identified and can be exemplified by strains causing mastitis in cattle. Whole genome sequence analysis of cattle-associated strains belonging to CC133 and CC151 has revealed evidence of genomic adaptations towards the bovine host, more specifically the bovine udder environment [2], [3]. *S. aureus* lineages associated with other animal species have also been described such as CC5 which is highly prevalent among poultry and CC8 that is commonly isolated from horses [4], [5].

Animal-associated isolates of *S. aureus* that are methicillin-resistant are of both veterinary and medical interest and have been the subject of extensive research over the past 5 years. The MRSA ST398 emerged as primarily a pig-associated strain, which has been found to be highly prevalent among pig herds in several countries in Europe and North America [6]–[9]. However, MRSA ST398 has also been isolated from other animal species such as cattle, poultry and horses [10]–[12]. Transfer of MRSA ST398 between animals and humans has been reported but the strain has also been isolated from persons with no history of livestock contact [13]–[15]. This exemplifies a probable lack of host specificity of this *S. aureus* strain. Nevertheless, the apparent recent emergence and rapid dissemination of MRSA ST398 among food-producing animals raises the question of what factors might have mediated the success of this lineage as livestock coloniser. Factors associated with pig husbandry such as movement of animals between different types of pig farms, use of antimicrobials and contact between animals and colonized personnel/veterinarians have been shown to be significant in occurrence and transmission of MRSA ST398 [16]. However, the molecular characteristics of ST398 should also be considered. The genomic analysis of various clonal complexes demonstrated clearly that *S. aureus* is an evolving pathogen and that around 22% of *S. aureus* genome consists of strain-specific elements that can mediate host or niche adaptation [17]. Identification of genetic markers that distinguish the ST398 lineage might help in determining and understanding the mechanisms behind MRSA ST398 capacity for livestock colonisation.

In this study *S. aureus* isolates consisting of MRSA ST398 as well as MSSA and MRSA belonging to other ST/CC prevalent among either humans or animals, were characterised for virulence and antimicrobial resistance gene profiles. The comparator panel consisted of: ST5, ST8, ST15, ST22, CC30, CC97, CC130 and CC151. Strains belonging to ST5 have been previously reported as common among community acquired (CA) isolates [18] whereas ST15 has been reported to represent a successful lineage among CA- and hospital-acquired (HA) MSSA isolates [19]–[21]. ST22 and CC30 represent the two dominant HA-MRSA lineages that have become epidemic in the UK hospitals [22] whereas CC97, CC130 and CC151 represent predominant bovine-specific lineages [23]–[28]. ST8 is a successful lineage of both CA- and HA-MRSA [29] although in this study the ST8 group consisted of human MSSA and horse MRSA. The aim of the study was to conduct inter-lineage comparative analysis to determine whether MRSA ST398 strains carry any lineage-specific molecular markers. We also wanted to investigate the similarities and variations in virulence and antimicrobial resistance genotypes between ST398 and other analysed ST/CC to determine if MRSA ST398 shares any significant similarities with other prevalent lineages of *S. aureus.*

## Results

### Genetic Background of the Strains

The main genotypic features of isolates (*spa* type, multilocus sequence type, clonal complex and SCC*mec* type) are summarised in Table 1. 33 *spa* types and 13 MLST were detected. *Spa* type t011 was the most prevalent among MRSA ST398 isolates (n = 10). t034 and t567 were identified in 3 isolates each, with t1451 and t4872 detected in single isolate only. Considerable *spa* type variation was observed for MSSA isolates belonging to ST5, ST15, CC30 and CC97 where each *spa* type was detected in no more than two isolates. All human ST8 isolates carried *spa* type t008 whereas both animal isolates were t064. The majority of ST22 isolates belonged to *spa* type t032 (n = 4) and all but one of CC130 isolates were t843 (n = 3). All CC151 isolates carried *spa* type t529. The most prevalent SCC*mec* among MRSA strains was type IV (n = 20), which was detected in all ST8, ST22 and CC30 MRSA strains as well as majority of ST398. SCC*mec* type V was also detected but only among ST398 (n = 6). Three ST398 strains were non-typeable (NT).

**Table 1:** Genotypic features of strains characterised in this study.

No	ID	Host	*spa*	ST^a^	CC^b^	*mecA* c	SCC*mec* d
1	25	Human	t002	5	5	–	–
2	8	Human	t179	5	5	–	–
3	17	Human	t442	5	5	–	–
4	19	Human	t442	–	(5)	–	–
5	6	Human	t008	8	8	–	–
6	11	Human	t008	–	(8)	–	–
7	16	Human	t008	–	(8)	–	–
8	200	Horse	t064	8	8	+	IVd
9	201	Horse	t064	–	(8)	+	IVd
10	10	Human	t084	15	15	–	–
11	21	Human	t084	–	(15)	–	–
12	23	Human	t228	15	15	–	–
13	60	Human	t360	15	15	–	–
14	24	Human	t491	15	15	–	–
15	38	Human	t020	22	22	+	IVh
16	44	Human	t032	22	22	+	IVh
17	28	Human	t032	–	(22)	+	IVh
18	32	Human	t032	–	(22)	+	IVh
19	40	Human	t032	–	(22)	+	IVh
20	39	Human	t379	22	22	+	IVh
21	18	Human	t608	22	22	–	–
22	20	Human	unknown	22	22	–	–
23	5	Human	t012	30	30	–	–
24	13	Human	t012	–	(30)	–	–
25	34	Human	t018	36	30	+	IVh
26	65	Human	t018	–	(30)	+	IV
27	51	Human	t019	30	30	–	–
28	62	Human	t021	30	30	–	–
29	1	Human	t122	30	30	–	–
30	31	Human	t1675	36	30	+	IV
31	174	Cattle	t131	1527	97	–	–
32	189	Cattle	t224	97	97	–	–
33	198	Cattle	t224	–	(97)	–	–
34	177	Cattle	t267	118	97	–	–
35	197	Cattle	t267	–	(97)	–	–
36	190	Cattle	t359	97	97	–	–
37	195	Cattle	t359	–	(97)	–	–
38	175	Cattle	t521	97	97	–	–
39	173	Cattle	t6220	130	130	+	–
40	178	Cattle	t843	1245	130	+	–
41	181	Cattle	t843	–	(130)	+	–
42	182	Cattle	t843	–	(130)	+	–
43	179	Cattle	t529	1074	151	–	–
44	193	Cattle	t529	–	(151)	–	–
45	194	Cattle	t529	–	(151)	–	–
46	199	Cattle	t529	–	(151)	–	–
47	90	Chicken	t011	398	398	+	IVc
48	91	Chicken	t011	–	(398)	+	V 5C2&5
49	92	Chicken	t011	–	(398)	+	IVa
50	99	Horse	t011	–	(398)	+	IVa
51	102	Rat	t011	–	(398)	+	IVa
52	103	Rat	t011	–	(398)	+	V 5C2&5
53	105	Cattle	t011	–	(398)	+	IVa
54	106	Cattle	t011	–	(398)	+	V 5C2&5
55	108	Pig	t011	–	(398)	+	V 5C2&5
56	109	Pig	t011	–	(398)	+	IVa
57	95	Human	t034	398	398	+	V 5C2&5
58	96	Human	t034	–	(398)	+	IVc
59	111	Pig	t034	–	(398)	+	IVa
60	101	Horse	t1451	398	398	+	IVa
61	104	Rat	t4872	398	398	+	V 5C2&5
62	93	Chicken	t567	398	398	+	NT
63	107	Cattle	t567	–	(398)	+	NT
64	110	Pig	t567	–	(398)	+	NT

a ‘-‘ not determined;

b determined or presumptive (based on *spa* type);

c ‘-‘ absent, ‘+’ present;

d ‘-‘ not applicable, NT: non-typeable.

### Superantigen Genes

**Figure 1:**
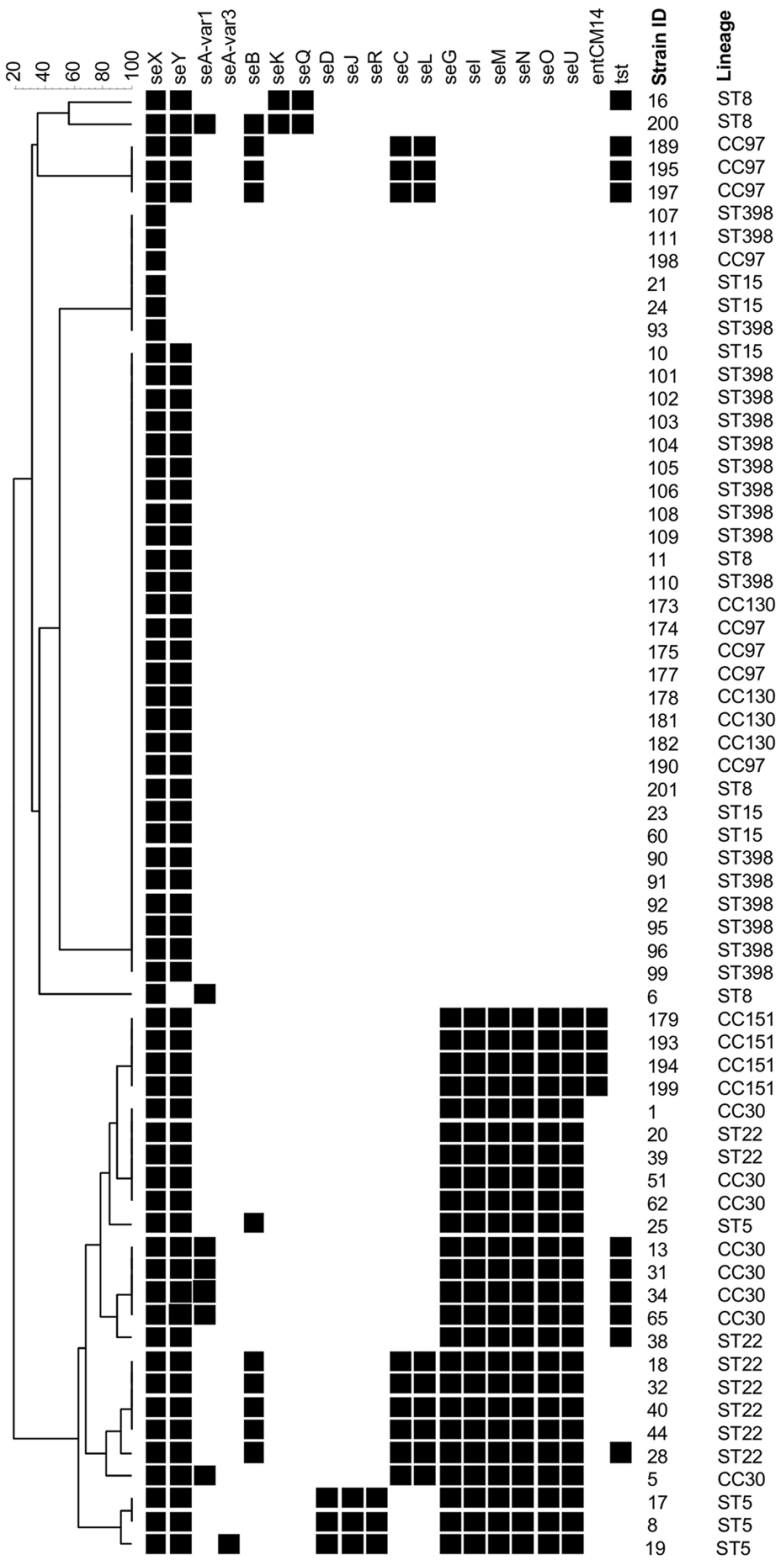
Superantigen gene profiles. UPGMA dendrogram displaying clustering and genetic similarity (using Jaccard’s coefficient) of superantigen gene profiles of analysed strains. Black box – positive; grey – ambiguous; white/blank – negative.

The carriage of enterotoxin genes is presented in Figure 1 and Table 2. Enterotoxin gene *seX* was detected in all analysed isolates while *seY* was identified in all analysed ST/CC but was absent in 7 isolates from various lineages (ST8, ST15, CC97 and ST398). Enterotoxin A gene *seA* was detected at low prevalence in three ST/CC: ST5 (n = 1), ST8 (n = 2), CC30 (n = 5). The *seB*+*seK*+*seQ* enterotoxin gene cluster was detected in a single ST8 isolate, with another ST8 isolate carrying the *seK*+*seQ* only. Furthermore, several isolates belonging to various lineages were positive for *seB* only: ST5 (n = 1), ST22 (n = 5), CC97 (n = 3). The *seD*+*seJ*+*seR* cluster was identified in all but one ST5 isolates and no other ST/CC group whereas the *seC*+*seL* genes were present in 9 isolates belonging to ST22 (n = 5), CC30 (n = 1) and CC97 (n = 3). The *egc* cluster: *seG*+*seI*+*seM*+*seN*+*seO*+*seU* was prevalent among isolates belonging to ST5, ST22, CC30 and CC151 but absent from all other ST/CC. The *entCM14* gene was present in all CC151 isolates and no other lineage. Finally, the *tst* gene was variably detected in several ST/CC: ST8 (n = 1), ST22 (n = 2), CC30 (n = 4), and CC97 (n = 3). All isolates were negative for *seE* and *seH*. The ST398 isolates carried no accessory enterotoxin genes, which was also observed for all ST15 and CC130 isolates.

**Table 2:** Comparison of the distribution of selected virulence genes/gene clusters that were detected at high frequency among some of the analysed lineages.

	Number of positive isolates								
	ST398	ST5	ST8	ST15	ST22	CC30	CC97	CC130	CC151
	(n = 18)	(n = 4)	(n = 5)	(n = 5)	(n = 8)	(n = 8)	(n = 8)	(n = 4)	(n = 4)
*seD+seJ+seR*	0	3	0	0	0	0	0	0	0
*seC+seL*	0	0	0	0	5	1	3	0	0
*egc*	0	4	0	0	8	8	0	0	4
*entCM14*	0	0	0	0	0	0	0	0	4
*lukF-PV-P83/lukM*	0	0	0	0	0	0	0	0	4
*lukD/E+splA+splB*	0	4	3	4	0	0	8	0	4
*hlb*	9	0	2	0	1	0	6	4	4
*sak*	0	4	3	0	7	8	0	0	0
*hl-III*	18	4	5	5	0	8	6	0	0
*edinB*	0	0	0	0	0	0	0	4	0

### *agr* Locus and *set* Genes

**Figure 2:**
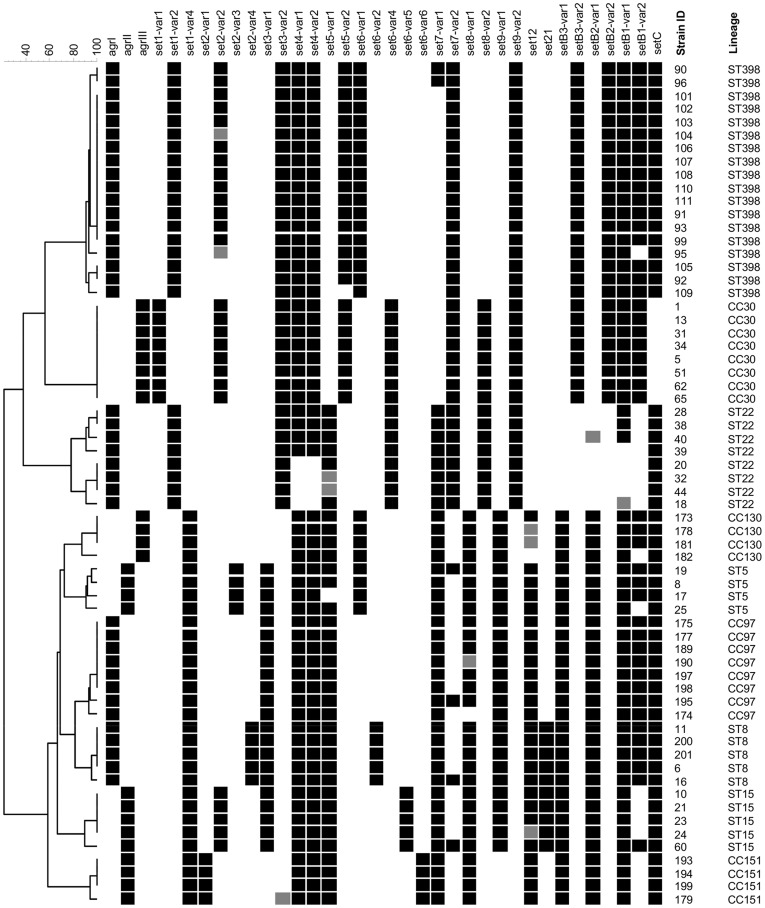
*agr* and *set* gene profiles. UPGMA dendrogram displaying clustering and genetic similarity (using Jaccard’s coefficient) of *agr* and *set* gene profiles of analysed strains. Black box – positive; grey – ambiguous; white/blank – negative.

The carriage of *set* genes and *agr* type is presented in Figure 2. Strains belonging to the same lineage displayed an identical accessory gene regulator (*agr*) type: ST8, ST22, CC97 and ST398 harboured *agrI*; ST5, ST15 and CC151 displayed *agrII*; whereas CC30 and CC130 contained *agrIII*. The *set* genes encode staphylococcal exotoxin-like proteins. The inter-lineage variation for *set* carriage can be defined by two factors: presence/absence of a particular gene and the allelic variant. The following genes: *set1*, *set4*, *set5* and *set7* were detected in all ST/CC but the allelic variant differed between lineages (all *set4*-positive isolates are displayed to carry both *set4-var1* and *set4-var2* as due to a strong cross-reactivity of the probes the true positive variant could not be identified). Other frequently identified *set* genes were also: *set3* (not detected in CC130), *set6* (not detected in CC97), *set8* (not detected in ST398), *set9* (not detected in CC151). For *set6*, the majority of isolates were found to carry one of four of the previously described allelic variants. Isolates belonging to ST15 and CC151 displayed a distinct combination of probes, different for both lineages, and were termed *var5* and *var6*, respectively. Common but less prevalent were also: *set2* (detected in ST5, ST8, ST15, CC30, CC151 and ST398) and *set12* (detected in ST5, ST8, ST15, CC97, CC130 and CC151). The *set21* gene was detected only in ST8 and ST15. A complete *setB* gene cluster, which consists of *setB3*, *setB2* and *setB1*, was detected in all ST/CC except ST22. The *setC* gene was also highly prevalent and could be detected in all isolates apart from those belonging to CC30 group.

### Leukocidin, Haemolysin and Other Virulence Genes

**Figure 3:**
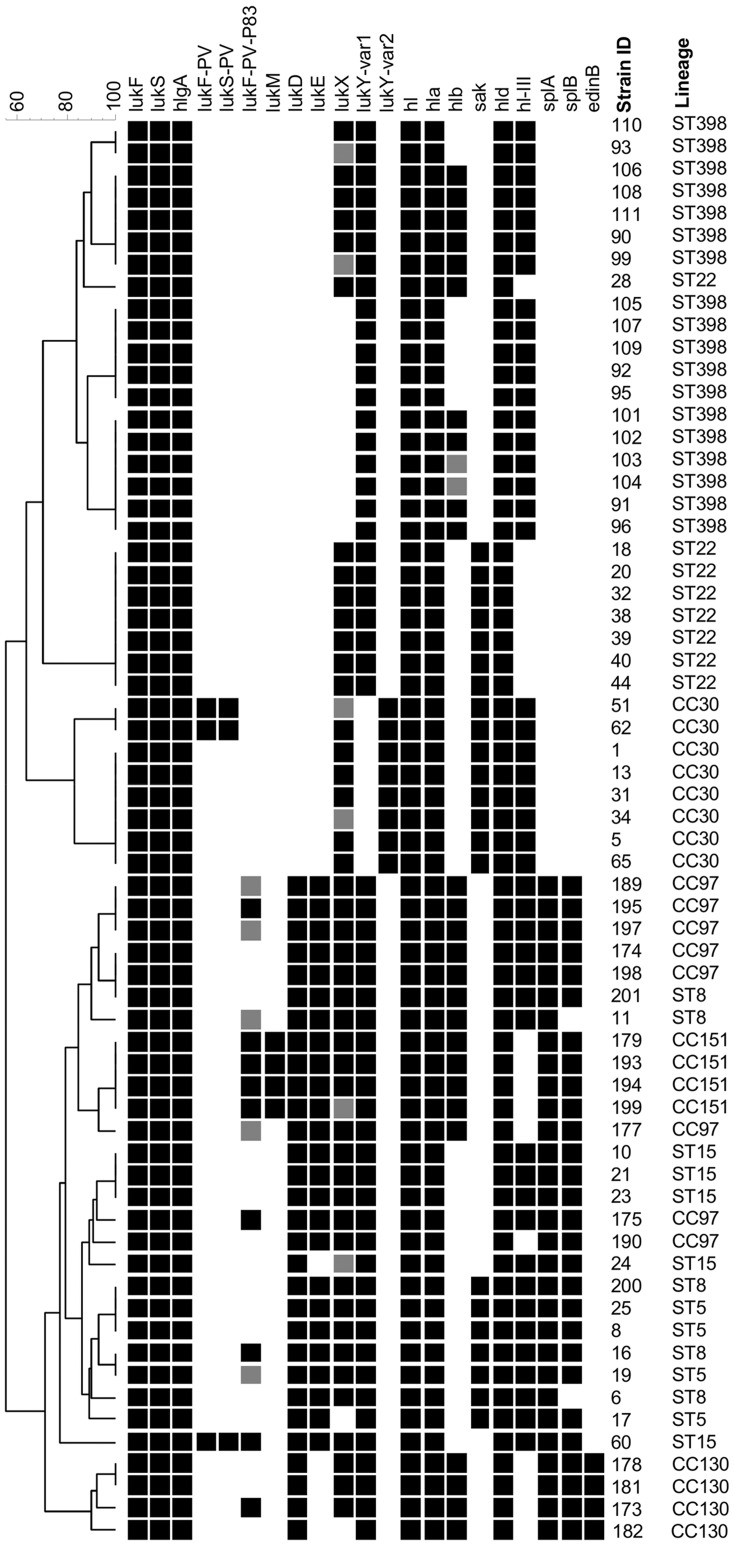
Leukocidin, haemolysin and other virulence gene profiles. UPGMA dendrogram displaying clustering and genetic similarity (using Jaccard’s coefficient) of leukocidin, haemolysin and other virulence gene profiles of analysed strains. Black box – positive; grey – ambiguous; white/blank – negative.

The carriage of leukocidin, haemolysin and other virulence genes is presented in Figure 3 and Table 2. All isolates carried *lukF*/*S*, *hlgA*, *hl*, *hla*, *hld* and *lukY* genes. *lukX* was detected in all ST/CC but not all analysed isolates. The *lukD+lukE*, *splA* and *splB* genes, known to reside together on an allelic variant of Saβ genomic island, were all detected in all or majority of isolates belonging to ST5, ST8 (two isolates *splB* negative), ST15 (one isolate *lukE* negative), CC97 and CC151. The genes, except for *lukE,* were also detected among CC130 isolates. The *lukF-PV-P83/lukM* genes were detected in all CC151 isolates and no other lineage. However, the *lukF-PV-P83* only was variably detected among isolates belonging to ST5, ST8 and CC97. The PVL leukocidin genes *lukF/S-PV* were identified only among isolates belonging to ST15 (n = 1) and CC30 (n = 2). An intact *hlb* gene (not disrupted by *hlb*-converting bacteriophage) was present in all isolates belonging to CC130 and CC151, as well as in isolates belonging to ST8 (n = 2), ST22 (n = 1), CC97 (n = 5) and ST398 (n = 9). Among *hlb*-negative isolates the *sak* gene was detected in over 60% (22/36), mainly those belonging to ST5 (n = 4), ST22 (n = 7) and CC30 (n = 8). The *hl-III* haemolysin gene was found in all isolates belonging to ST5, ST8, ST15, CC30 and ST398 as well as majority of CC97 (n = 6). Finally *edinB* was identified among all CC130 isolates but was absent in all other analysed lineages. All isolates were negative for *etA, etB, etD, edinA* and *edinC.*

### Adhesion Genes

**Figure 4:**
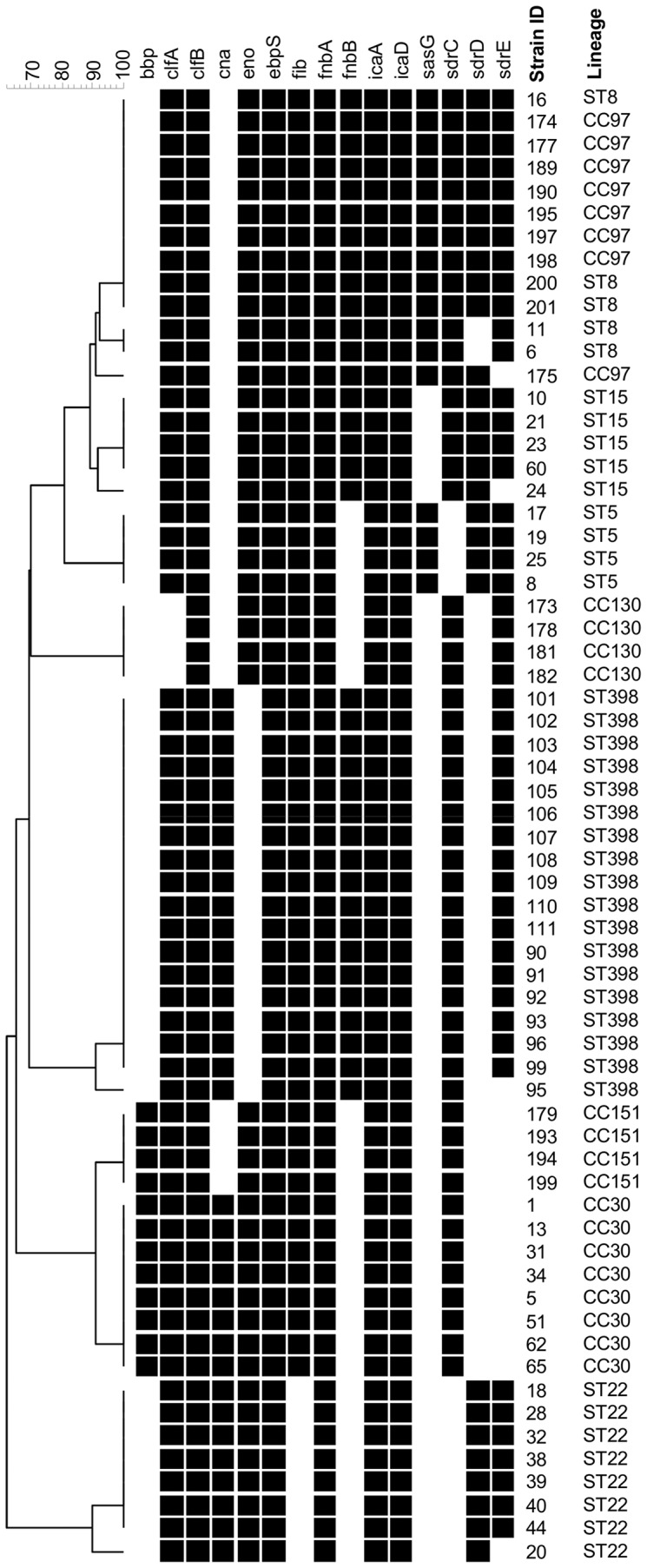
Adhesion gene profiles. UPGMA dendrogram displaying clustering and genetic similarity (using Jaccard’s coefficient) of adhesion gene profiles of analysed strains. Black box – positive; grey – ambiguous; white/blank – negative.

The carriage of adhesion genes is presented in Figure 4. The genes: *clfB*, *ebpS*, *fnbA*, *icaA* and *icaD* were identified in all isolates. Other highly prevalent adhesion genes were: *clfA* (not detected in CC130), *eno* (not detected in ST398), *fib* (not detected in ST22), *sdrC* (not detected in ST5 and ST22) and *sdrE* (not detected in CC30 and CC151). Less common but also detected among analysed lineages were: *bbp* (detected in CC30 and CC151), *cna* (detected in ST22, CC30 and ST398), *fnbB* (detected in ST8, ST15, CC97 and ST398), *sasG* (detected in ST5, ST8 and CC97) and *sdrD* (detected in ST5, ST8, ST15, ST22 and CC97). The PCR detection of adhesion genes was specific and single bands only of expected size were observed.

### Antimicrobial Resistance Phenotypes and Genotypes

**Figure 5:**
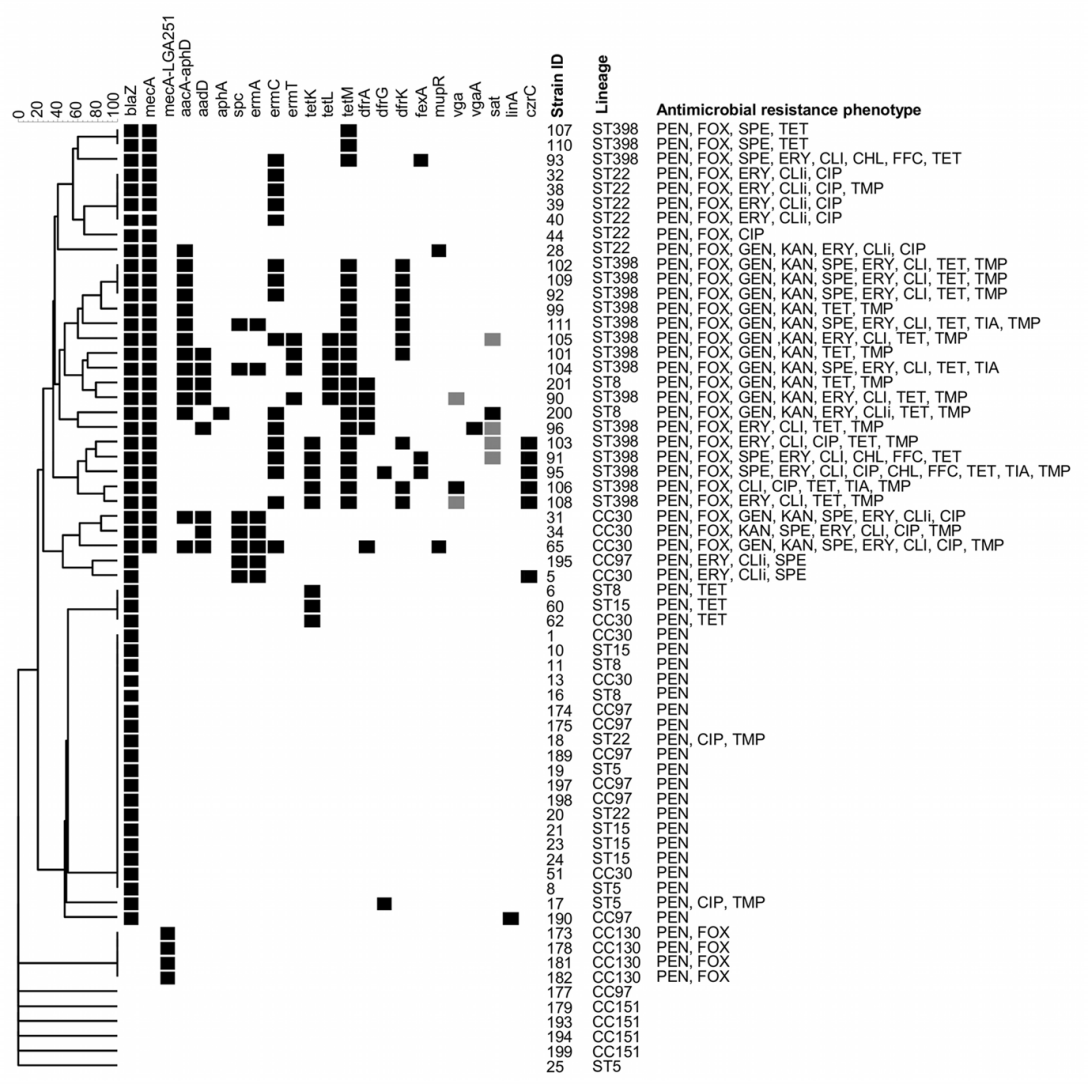
Antimicrobial resistance genotypes and phenotypes. UPGMA dendrogram displaying clustering and genetic similarity (using Jaccard’s coefficient) of antimicrobial resistance gene profiles of analysed strains. Black box – positive; grey – ambiguous; white/blank – negative. The far right column shows antimicrobial resistance phenotype. PEN, penicillin; FOX, cefoxitin; GEN, gentamicin; KAN, kanamycin; SPE, spectinomycin; CLI, clindamycin; ERY, erythromycin; CIP, ciprofloxacin; CHL, chloramphenicol; FFC, florfenicol; TET, tetracycline; TIA, tiamulin; TMP, trimethoprim; i, inducible.

The results are presented in Figure 5 and Table 3. The majority of analysed isolates displayed penicillin resistance (n = 58). Methicillin resistance was detected for all isolates belonging to ST398 and CC130 as well as among equine ST8 (n = 2), ST22 (n = 6) and CC30 (n = 3) isolates. All ST398 isolates also displayed resistance to tetracycline, which was also detected among ST8 (n = 3), ST15 (n = 1) and CC30 (n = 1). Gentamicin-kanamycin resistant isolates included ST398 (n = 9), equine ST8 (n = 2), ST22 (n = 1) and CC30 (n = 2). A single isolate belonging to CC30 was kanamycin-resistant but gentamicin susceptible. Spectinomycin resistance was detected among ST398 (n = 10), CC30 (n = 4) and CC97 (n = 1). Erythromycin-clindamycin resistant isolates consisted of ST398 (n = 13), equine ST8 (n = 1), ST22 (n = 5), CC30 (n = 4) and CC97 (n = 1). One other isolate belonging to ST398 was clindamycin-resistant but susceptible to erythromycin. Resistance to trimethoprim was identified among ST398 (n = 13), ST5 (n = 1), equine ST8 (n = 2), ST22 (n = 2) and CC30 (n = 2). Chloramphenicol-florfenicol as well as tiamulin resistance was detected among ST398 isolates only (n = 3, n = 4, respectively). Resistance to ciprofloxacin was detected among ST398 (n = 3), ST5 (n = 1), ST22 (n = 7) and CC30 (n = 3). All isolates were susceptible to apramycin, vancomycin and teicoplanin.

**Table 3:** Comparison of the distribution of selected resistance genes among MRSA strains analysed in this study.

	Number of positive isolates			
	ST398	ST8	ST22	CC30
	(n = 18)	(n = 2)	(n = 6)	(n = 3)
*aacA-aphD*	9	2	1	2
*aadD*	4	1	0	3
*aphA*	0	1	0	0
*spc*	2	0	0	3
*ermA*	2	0	0	3
*ermC*	10	1	4	1
*ermT*	4	0	0	0
*tetK*	5	0	0	0
*tetL*	4	1	0	0
*tetM*	18	2	0	0
*dfrA*	2	2	0	1
*dfrG*	1	0	0	0
*dfrK*	10	0	0	0

The *blaZ* gene was detected in 84% of analysed isolates. The *blaZ*-negative isolates included all CC130 and CC151 strains. The *mecA* gene was identified in all ST398 strains as well as among isolates belonging to ST8 (n = 2), ST22 (n = 6) and CC30 (n = 3). All CC130 strains carried the *mecA* homologue - *mecA*_LGA251_. The *aacA*-*aphD* was the most common aminoglycoside resistance gene and it was detected in various lineages: ST398 (n = 9), ST8 (n = 2), ST22 (n = 1) and CC30 (n = 2). The *aadD* gene was found in ST398 (n = 4), ST8 (n = 1) and CC30 (n = 3) with all but two of these isolates also carrying the *aacA-aphD*. Single isolate belonging to ST8 carried the *aphA* gene (also *aacA-aphD* positive). The erythromycin resistance gene *ermA* was mainly detected in isolates belonging to CC30 (n = 4) but was also found among ST398 (n = 2) and CC97 (n = 1). All *ermA*-positive isolates carried also the spectinomycin resistance gene *spc*. More common among ST398 isolates was the *ermC* gene (n = 10), also detected in ST8 (n = 1) and ST22 (n = 4). The *ermT* gene was detected among ST398 isolates only (n = 4). All ST398 strains were positive for the *tetM* gene, which was also detected in equine ST8 (n = 2) isolates. The *tetK* gene was detected among ST398 (n = 5), ST8 (n = 1), ST15 (n = 1) and CC30 (n = 1). Furthermore, the *tetL* gene was found in ST398 (n = 4) as well as ST8 (n = 1). The trimethoprim resistance genes were prevalent mainly among ST398 with 55% of isolates carrying the *dfrK* gene (n = 10), which was not detected in any other lineage. The *dfrA* was identified in ST398 (n = 2), ST8 (n = 2) and CC30 (n = 1). The *dfrG* gene was detected in two isolates, one belonging to ST398 and other to ST5. The chloramphenicol-florfenicol resistance gene *fexA* was detected among ST398 isolates only (n = 3). The *czrC*, cadmium and zinc resistance gene, recently found to be associated with the SCC*mec* element from MRSA ST398, was identified in all but one ST398 isolates carrying the SCC*mec* type V as well as in a single MSSA isolate belonging to CC30. The detection of resistance genes by PCR was specific and single bands only of expected size were observed. Ambiguous DNA microarray results were obtained for *vga* and *sat* genes among some ST398 strains.

In summary, the MRSA ST398 isolates displayed a considerable antimicrobial resistance genotype heterogeneity as well as diversity. This feature was not, however, strictly restricted to the ST398 lineage. High content of antimicrobial resistance genes could also be observed in MRSA strains belonging to ST8 and CC30, with MRSA ST22 displaying a comparatively lower prevalence of resistance determinants.

## Discussion

The DNA microarray-based analysis revealed a limited content of virulence determinants among MRSA ST398 isolates in comparison to the representatives of other ST/CC included in this study. The absence of significant virulence factors among isolates belonging to ST398 has been previously observed and later confirmed by the whole genome analysis of a MRSA ST398 strain [12], [30], [31]. The majority of virulence genes that were detected among ST398 strains were common among all ST/CC and represent the core virulence genes of *S. aureus*. We identified several genes that, although not unique to the ST398 lineage, were not prevalent among all analysed lineages and thus might mediate the ST398 ability for enhanced animal colonisation.

An intact *hlb*, which encodes a beta-haemolysin, was detected in 50% of ST398 isolates as well as in all or majority of cattle-associated isolates belonging to CC97, CC130 and CC151. The gene is chromosomally located but might become inactivated by insertion of *hlb*-converting phages that carry immune evasion genes such as *sak*, *chp* and *scn* [32]–[35]. Although these elements can provide the strain with a selective advantage, they encode human-specific molecules [35]. As such, the carriage of staphylokinase gene *sak* is prevalent among human strains whereas the presence of an intact *hlb* is common among cattle isolates [36], [37]. In this study all isolates carrying an intact *hlb* were negative for *sak*, confirming that the presence of an intact *hlb* gene and carriage of *sak* is commonly antagonistic. Some of the isolates belonging to ST15, CC97 and ST398 carried a disrupted *hlb* but were *sak*-negative, which might indicate an insertion of a different phage. Nevertheless the failure to detect *sak* among any of the ST398 isolates and presence of an intact *hlb* gene could suggest a lack of adaptation for the human host and/or mechanism for animal-specific colonisation.

Two of the adhesion genes detected among ST398 isolates displayed limited prevalence among other ST/CC: *fnbB* and *cna*. The *fnbB* gene is a homologue of *fnbA* and both genes encode fibronectin-binding protein. Although *fnbA* is highly prevalent and was identified in all analysed isolates, the *fnbB* gene was only detected in ST8, ST15, CC97 and ST398. It has been shown that the presence of both *fnbA* and *fnbB* can significantly enhance biofilm formation in MRSA isolates [38]. The importance of *fnbA*+*fnbB* carriage among ST398 isolates is unclear in the context of animal-specific colonisation as the cooperative function of *fnbA*- and *fnbB*-expressed molecules was shown to be significant for the development of systemic infection [39]. Of higher significance might be the carriage of collagen adhesion gene *cna* by all ST398 isolates. It was detected in only two other lineages, namely ST22 and CC30. The *cna* gene was previously reported as prevalent among pandemic CA-MRSA isolates belonging to ST30 [40]. It was also suggested that the gene might play role in facilitating stable colonisation [41].

The analysis of lineages other than ST398 revealed a probable association between certain virulence genes and specific ST/CC. The *lukF-PV-P83*/*lukM* locus, which encodes a bi-component leukotoxin that is highly active against bovine neutrophils, was previously reported to be common among cattle isolates related to the RF122 strain [42]. Similarly, in this study the *lukF-PV-P83*/*lukM* locus was detected only among CC151 isolates. The enterotoxin gene cluster *egc* was found among all ST5, ST22, CC30 and CC151 strains. This cluster has been previously reported to be more common in carriage strains than invasive isolates and to be prevalent among nasal isolates [41], [43]. In this study such association could not be determined; instead the *egc* cluster appears to be prevalent among specific *S. aureus* lineages. Our data also shows that the allelic variant of staphylococcal genomic island vSaβ carrying *splA*, *splB* and *lukD*/*lukE* genes might be prevalent in isolates belonging to ST5, ST8, ST15, CC97, CC130 and CC151.

We found a varying level of similarity in virulence genotypes between the ST398 lineage and other ST/CC. The comparison of superantigen genotypes revealed that the lack of enterotoxin genes is not a unique feature of ST398 as the same profile was identified for all isolates belonging to ST15 and CC130. Comparing genes associated with leukocidins, haemolysins and serine proteases shows that ST398 isolates most closely resemble the ST22 and CC30 groups as all lack *lukD/lukE*, *splA* and *splB* genes. These three lineages also displayed similar *set* gene profiles. Interestingly, both ST22 and ST398 lineages were found to carry CC30-specific allelic variants of some of the *set* genes. The exact function of *set*-encoded exotoxin-like proteins remains to be determined but it has been demonstrated that the *set* products exhibit immunostimulatory properties [44]. Identification of *set* markers that are shared between ST398 and other successful MRSA lineages such as ST22 and CC30 can provide candidate genes for further investigation of individual *set* genes and their role in host colonisation and infection.

The clustering of antimicrobial resistance genotypes was highly heterogeneous and profile similarity was observed for strains belonging to different ST/CC. It has been previously observed by Monecke *et al* [42] that the carriage of antibiotic resistance determinants is largely non-lineage specific due to the promiscuous nature of mobile genetic elements carrying them. Interestingly, the resistance profile of ST398 was most comparable to the two equine MRSA ST8 strains. They shared the *aacA-aphD, aadD, ermC, tetL* and *dfrA* genes with the ST398 isolates and most importantly both ST8 isolates carried *tetM*. This suggests that the animal-associated isolates share a common resistance gene pool amongst each other to a greater degree than with the human strains. Evaluation of a larger collection of animal *S. aureus* strains, both MSSA and MRSA would be necessary to further investigate this finding. Moreover, the diversity and high prevalence of *tet* genes might not be a unique feature of MRSA ST398 but rather a common characteristic of animal-associated MRSA.

In conclusion, to date it is unclear what factors mediate the rapid dissemination of MRSA ST398 among livestock. The failure to identify any significant virulence factors in MRSA ST398 strains might suggest that the lineage harbours novel virulence determinants, particularly those that might enhance colonisation. Although the ST398 resistance gene pool is likely to have played a significant role in the selection of MRSA ST398 as livestock-coloniser, we have demonstrated in this study that similar antimicrobial resistance gene profiles can be detected among other animal-associated MRSA strains such as ST8. Further investigations into MSSA and MRSA isolates from different animal species are necessary to confirm the relative contribution the resistance genes and virulence determinants encoding colonisation factors have played in the spread of successful clones, as well as the relative importance of such factors in comparison with animal husbandry and movement factors.

## Materials and Methods

### Bacterial Strains

A total of sixty four *S. aureus* isolates of human and animal origin were investigated (Table 1). Each isolate analysed in this study was selected on the basis of representing a unique fingerprint as determined by pulsed-field gel electrophoresis (Figures S1, S2) following an analysis of a panel of 134 isolates and using a cut-off value of 100% for differentiation of pulsotypes. The PFGE was conducted as described by the Harmony protocol [45]. All ST398 isolates were non-typeable with *SmaI* and the genomic DNA was successfully digested with *Cfr9I*.

All isolates belonging to ST398 (n = 18) were isolated in Belgium from various host species: human (pig farmers, n = 2), pig (surveillance, n = 4), cattle (mastitis, n = 3), horse (infection, n = 2) chicken (surveillance, n = 4) and rat (surveillance, n = 3) between 2008 and 2010. The non-ST398 comparator panel consisted of human and animal isolates. Cattle isolates (n = 16) were isolated from cases of clinical mastitis in the UK from April 2006 to September 2007. Equine clinical isolates (n = 2) were isolated in Northern Ireland in 2010. Human isolates (n = 47) were isolated in the UK in the London area from Kingston Hospital, The Royal Brompton Hospital, and from The Royal Marsden Hospital, between 2007 and 2009.

### Molecular Typing

Isolates were *spa*-typed [46], [47] and sequence type was determined by multilocus sequence typing (MLST) for one representative of each *spa* type identified [48]. *Spa* type, MLST and clonal complexes were assigned using BioNumerics software (Version 5.10; Applied Maths, Belgium). SCC*mec* typing was conducted according to the method of Boye *et al* [49]. When SCCmec type IV was identified the strains were sub-typed using the method developed by Milheirico *et al* [50] whereas strains harbouring SCC*mec* type V were sub-typed according to method by Higuchi *et al* [51].

### DNA Microarray Analysis

DNA microarray analysis was performed using Identibac MRSA Array-Tube (AT, STAU-PM5_5, production year: 2008; Identibac, Surrey, UK). Genomic DNA was isolated using Clondiag StaphyType Kit lysis reagents and Qiagen DNeasy kit, following the StaphyType protocol (StaphyType; Alere Technologies GmbH, Germany). Amplification, labelling and array hybridisation of target DNA was performed according to the manufacturer’s instructions (Identibac, Surrey, UK). In short, around 1.5 µg of the DNA was amplified using primers provided by the manufacturer and labelled by incorporation of biotin-16-dUTP (Roche Diagnostics, Germany). The labelled PCR product was hybridised to the probes, after which the AT was washed successively with 2×SSC 0.01% Triton, 2×SSC and 0.2×SSC buffers, followed by treatment with 2% blocking solution and addition of poly-HRP streptavidin (Thermo Fisher Scientific Inc, USA). The AT was washed again and peroxidise substrate was added (Seramun green; Seramun, Germany). The chip images were captured using ATR03 reader (AlereTechnologies GmbH, Germany). Visualisation and analysis of hybridized probes was conducted using IconoClust software package (CLONDGIAG; Germany). The analysis of raw data (probe signal values, Table S1) was performed in accordance with previously published methods [36], [42]. The results were converted into numerical format (2– present, 1– ambiguous, 0– absent) and further analysed using BioNumerics (Version 5.10; Applied Maths, Belgium).

### PCR Analysis of Adhesion and Resistance Genes not Present on the Array

Isolates were also screened by PCR for the presence of adhesion and additional resistance genes using previously described primers and thermal cycling conditions (Table S2). The selected adhesion genes tested were: *bbp, cna, eno, ebpS, fnbB, fib, clfA*, *clfB*, *fnbA*, *sdrC, sdrD*, *sdrE*, *icaA*, *icaD* and *sasG*. The additional resistance genes tested were: *tet*L, *dfr*K, *spc*, *erm*T, *czr*C and *mecA*_LGA251_. Gene was considered as present if the PCR product was a single and specific band of the expected length. The PCR results were converted into numerical format (2– present, 0– absent) and analysed using BioNumerics (Version 5.10; Applied Maths, Belgium). The adhesion gene profiles were based on PCR results only whereas the antimicrobial resistance genotypes consisted of collated DNA microarray and PCR data.

### Antimicrobial Susceptibility Testing

Antimicrobial susceptibility testing was performed by the broth microdilution method, using in-house prepared microtitre plates following CLSI recommendations [52]. The following antimicrobials were evaluated: penicillin G, cefoxitin, gentamicin, kanamycin, spectinomycin, apramycin, erythromycin, vancomycin, teicoplanin, clindamycin, ciprofloxacin, tetracycline, chloramphenicol, florfenicol, trimethoprim and tiamulin. Interpretation of MICs was according to the CLSI guidelines. *S. aureus* ATCC 29123 was used as a control strain.

## Supporting Information

Figure S1 **UPGMA cluster analysis of PFGE patterns (after** ***SmaI*** **digestion) using Dice similarity coefficient and position tolerance of 1.2%.**
(DOC)

Figure S2 **UPGMA cluster analysis of PFGE patterns (after** ***Cfr9I*** **digestion) using Dice similarity coefficient and position tolerance of 1.2%.**
(DOC)

Table S1 **Raw DNA microarray data showing signal intensity values for all array tube probes.**
(XLS)

Table S2 **Primers used in this study.**
(DOC)
