# Supplementary material for: Comparative Genotypic and Phenotypic Characterisation of Methicillin-Resistant Staphylococcus aureus ST398 Isolated from Animals and Humans
Source: PLoS One. 2012 Jul 11;7(7):e40458. doi: 10.1371/journal.pone.0040458 (PMC3394705; doi:10.1371/journal.pone.0040458)
Supplement: Figure S2 — UPGMA cluster analysis of PFGE patterns (after Cfr9I digestion) using Dice similarity coefficient and position tolerance of 1.2%. (DOC) [file pone.0040458.s002.doc]

Figure S2. UPGMA cluster analysis of PFGE patterns (after *Cfr9I* digestion) using Dice similarity coefficient and position tolerance of 1.2%.
