# Supplementary material for: Comparative Genotypic and Phenotypic Characterisation of Methicillin-Resistant Staphylococcus aureus ST398 Isolated from Animals and Humans
Source: PLoS One. 2012 Jul 11;7(7):e40458. doi: 10.1371/journal.pone.0040458 (PMC3394705; doi:10.1371/journal.pone.0040458)
Supplement: Table S2 — Primers used in this study. (DOC) [file pone.0040458.s004.doc]

**Table S2**. Primers used in this study

| Gene | Name | Sequence 5’-3’ | Size (bp) | Ref |
| --- | --- | --- | --- | --- |
| *bbp* | BBP-1  BBP-2 | AACTACATCTAGTACTCAACAACAG  ATGTGCTTGAATAACACCATCATCT | 575 | [1] |
| *cna* | CNA-1  CNA-2 | GTCAAGCAGTTATTAACACCAGAC  AATCAGTAATTGCACTTTGTCCACTG | 423 | [1] |
| *eno* | ENO-1  ENO-2 | ACGTGCAGCAGCTGACT  CAACAGCATYCTTCAGTACCTTC | 302 | [1] |
| *ebpS* | EBP-1  EBP-2 | CATCCAGAACCAATCGAAGAC  CTTAACAGTTACATCATCATGTTTATCTTTG | 186 | [1] |
| *fnbA* | fnbA_F  fnbA_R | CATAAATTGGGAGCAGCATCA  ATCAGCAGCTGAATTCCCATT | 127 | [2] |
| *fnbB* | FNBB-1  FNBB-2 | GTAACAGCTAATGGTCGAATTGATACT  CAAGTTCGATAGGAGTACTATGTTC | 524 | [1] |
| *fib* | FIB-1  FIB-2 | CTACAACTACAATTGCCGTCAACAG  GCTCTTGTAAGACCATTTTCTTCAC | 404 | [1] |
| *clfA* | CLFA-1  CLFA-2 | ATTGGCGTGGCTTCAGTGCT  CGTTTCTTCCGTAGTTGCATTTG | 292 | [1] |
| *clfB* | CLFB-1  CLFB-2 | ACATCAGTAATAGTAGGGGGCAAC  TTCGCACTGTTTGTGTTTGCAC | 205 | [1] |
| *sdrC* | sdrC_F  sdrC_R | ACGACTATTAAACCAAGAAC  GTACTTGAAATAAGCGGTTG | 560 | [3] |
| *sdrD* | sdrD_F  sdrD_R | GGAAATAAAGTTGAAGTTTC  ACTTTGTCATCAACTGTAAT | 500 | [3] |
| *sdrE* | sdrE_F  sdrE_R | CAGTAAATGTGTCAAAAGA  TTGACTACCAGCTATATC | 767 | [3] |
| *icaA* | AF  AR | CCTAACTAACGAAAGGTAG  AAGATATAGCGATAAGTGC | 1315 | [4] |
| *icaD* | DF  DR | AAACGTAAGAGAGGTGG  GGCAATATGATCAAGATAC | 381 | [4] |
| *sasG* | sasG_F  sasG_R | CGCGGATTCGCAGCTGAAAACAATATT  CCCAAGCTTTAATTCTGTTATTGTTTTTGG | 1110 | [5] |
| *tetL* | tetL_F  tetL_R | TCGTTAGCGTGCTGTCATTC  GTATCCCACCAATGTAGCCG | 267 | [6] |
| *dfrK* | dfrK_fw  dfrK_rv | GCTGCGATGGATAATGAACAG  GGACGATTTCACAACCATTAAAGC | 214 | [7] |
| *spc* | spc_fw  spc_rv | ACCAAATCAAGCGATTCAAA  GTCACTGTTTGCCACATTCG | 561 | [7] |
| *ermT* | ermT_fw  ermT_rv | ATTGGTTCAGGGAAAGGTCA  GCTTGATAAAATTGGTTTTTGGA | 536 | [7] |
| *czrC* | czrC_F  czrC_R | TAGCCACGATCATAGTCATG  ATCCTTGTTTTCCTTAGTGACTT | 655 | [8] |
| *mecA_LGA251_* | mecA-LGA_Fw  mecA-LGA_Rv | TCACCAGGTTCAACYCAAAA  CCTGAATCWGCTAATAATATTTC | 356 | [9] |

REFERENCES

1. Tristan A, Ying L, Bes M, Etienne J, Vandenesch F, et al. (2003) Use of multiplex PCR to identify *Staphylococcus aureus* adhesins involved in human hematogenous infections. J Clin Microbiol 41(9): 4465-4467.

2. Vancraeynest D, Hermans K, Haesebrouck F. (2004) Genotypic and phenotypic screening of high and low virulence *Staphylococcus aureus* isolates from rabbits for biofilm formation and MSCRAMMs. Vet Microbiol 103(3-4): 241-247.

3. Peacock SJ, Moore CE, Justice A, Kantzanou M, Story L, et al. (2002) Virulent combinations of adhesin and toxin genes in natural populations of *Staphylococcus aureus*. Infect Immun 70(9): 4987-4996.

4. CiftciI A, FindikI A, OnukII EE, Savasan S. (2009) Detection of methicillin-resistance and *slime* factor production of *Staphylococcus aureus* in bovine mastitis. Braz J Microbiol 40: 254-261.

5. Rohde H, Burandt EC, Siemssen N, Frommelt L, Burdelski C, et al. (2007) Polysaccharide intercellular adhesin or protein factors in biofilm accumulation of *Staphylococcus epidermidis* and *Staphylococcus aureus* isolated from prosthetic hip and knee joint infections. Biomaterials 28(9): 1711-1720.

6. Ng LK, Martin I, Alfa M, Mulvey M. (2001) Multiplex PCR for the detection of tetracycline resistant genes. Mol Cell Probes 15(4): 209-215.

7. Fessler A, Scott C, Kadlec K, Ehricht R, Monecke S, et al. (2010) Characterization of methicillin-resistant *Staphylococcus aureus* ST398 from cases of bovine mastitis. J Antimicrob Chemother 65(4): 619-625.

8. Cavaco LM, Hasman H, Stegger M, Andersen PS, Skov R, et al. (2010) Cloning and occurrence of *czrC*, a gene conferring cadmium and zinc resistance in methicillin-resistant *Staphylococcus aureus* CC398 isolates. Antimicrob Agents Chemother 54(9): 3605-3608.

9. Garcia-Alvarez L, Holden MT, Lindsay H, Webb CR, Brown DF, et al. (2011) Meticillin-resistant *Staphylococcus aureus* with a novel *mec*A homologue in human and bovine populations in the UK and Denmark: A descriptive study. Lancet Infect Dis 11(8): 595-603.
